# Supplementary material for: Hesperidin Is a Potential Inhibitor against SARS-CoV-2 Infection
Source: Nutrients. 2021 Aug 16;13(8):2800. doi: 10.3390/nu13082800 (PMC8401008; doi:10.3390/nu13082800)
Supplement: Supplementary file 1 [file nutrients-13-02800-s001.zip › nutrients-1269577-supplementary.pdf]

Hesperidin is a potential inhibitor against SARS-CoV-2 infection

Fang-Ju Cheng[1], Thanh Kieu Huynh[2], Chia-Shin Yang[3], Dai-Wei Hu[2], Yi-Cheng Shen[2,4], Chih-Yen Tu[4,5], Yang-Chang Wu[6], Chih-Hsin Tang[2,5], Wei-Chien Huang[1,2,7,8,*], Yeh Chen[1,3,*], Chien-Yi Ho[9, 10,*]

This PDF file includes:

Figure S1. The docking simulation of hesperetin in the pocket of human ACE2 bound to viral S protein.

Figure S2. The effect of HT/HD on the viability of VeroE6 cells.

Figure S3. The effect of HT and HD on the mRNA expressions of ACE2 and TMPRSS2.

Figure S4. Short term treatment with HT impedes the infection of SARS-CoV-2 pseudovirus into VeroE6 cells.

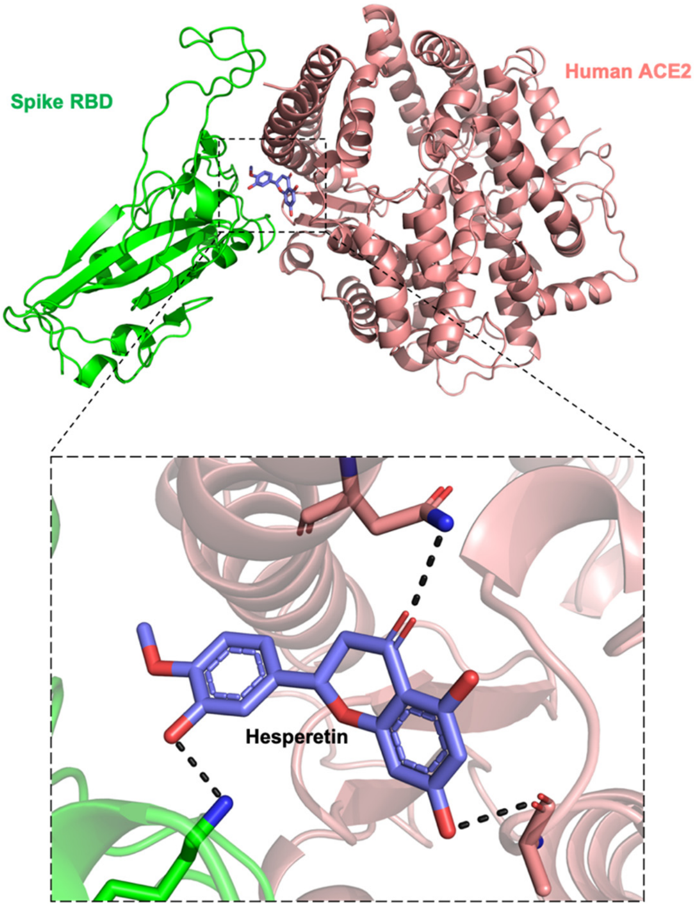

Figure S1. The docking simulation of hesperetin in the pocket of human ACE2 bound to viral S protein. Compound-protein interaction of HT with the site of ACE2 bound to S protein was generated by using Discovery Studio.

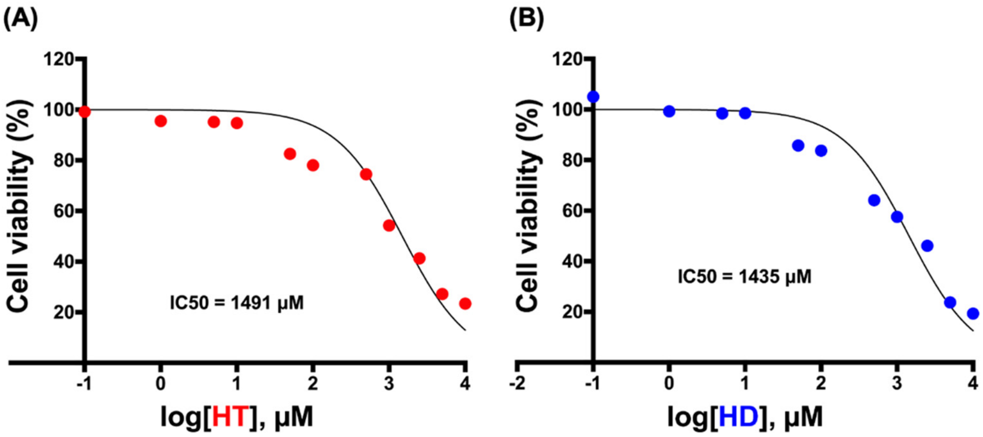

Figure S2. The effect of HT/HD on the viability of VeroE6 cells. VeroE6 cell line was treated with HT and HD in a dose-dependent manner for 2 days and the viability was determined in MTT assays.

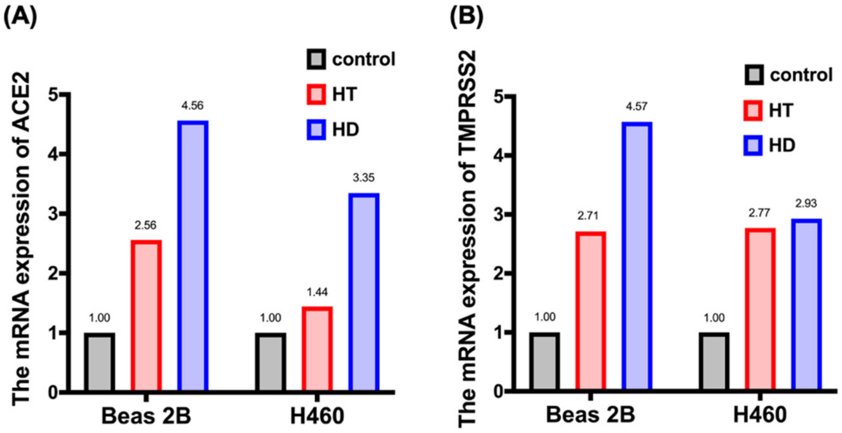

Figure S3. The effect of HT and HD on the mRNA expressions of ACE2 and TMPRSS2. Beas 2B and H460 cell lines were treated with 100 μM of HT or HD for 2 days, and the mRNA expressions of ACE2 (A) and TMPRSS2 (B) were examined in RT-qPCR analysis.

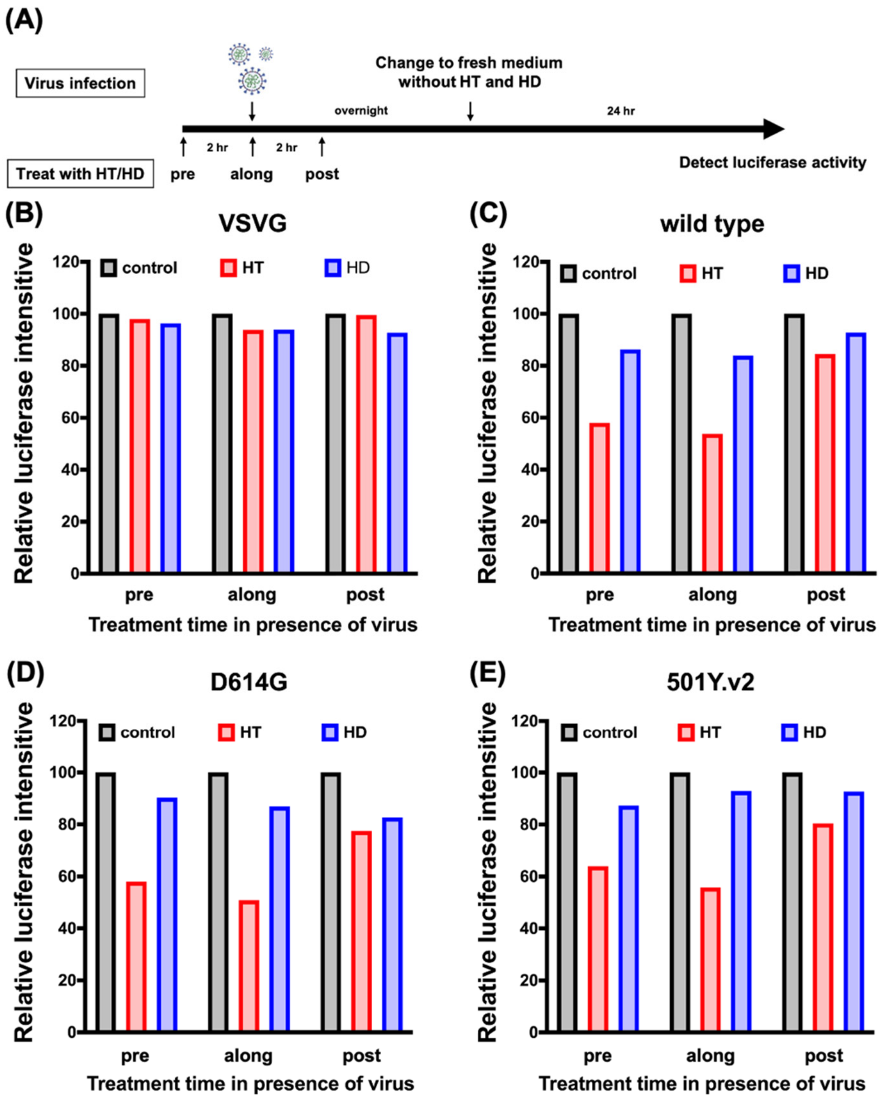

Figure S4. Short term treatment with HT impedes the infection of SARS-CoV-2 pseudovirus into VeroE6 cells. VeroE6 cells were treated with 100 μM of HT or HD 2 hours before (as indicated as "pre"), along (as indicated as "along"), or after (as indicated as "post") virus infections of VSVG pseudotyped vector (B), or SARS-CoV-2 pseudoviruses with wild type (C), D614G (D), and 501Y.v2 (E) of S protein followed by the measurement of luciferase activity.
